# Recognition of Emotional States Using Multiscale Information Analysis of High Frequency EEG Oscillations

**DOI:** 10.3390/e21060609

**Published:** 2019-06-20

**Authors:** Zhilin Gao, Xingran Cui, Wang Wan, Zhongze Gu

**Affiliations:** 1 Key Laboratory of Child Development and Learning Science, Ministry of Education, School of Biological Science & Medical Engineering, Southeast University, Nanjing 210000, China; 2 Institute of Biomedical Devices (Suzhou), Southeast University, Suzhou 215000, China

**Keywords:** emotion recognition, EEG, multiscale information analysis, multiscale sample entropy, ensemble empirical mode decomposition, fuzzy entropy, support vector machine

## Abstract

Exploring the manifestation of emotion in electroencephalogram (EEG) signals is helpful for improving the accuracy of emotion recognition. This paper introduced the novel features based on the multiscale information analysis (MIA) of EEG signals for distinguishing emotional states in four dimensions based on Russell’s circumplex model. The algorithms were applied to extract features on the DEAP database, which included multiscale EEG complexity index in the time domain, and ensemble empirical mode decomposition enhanced energy and fuzzy entropy in the frequency domain. The support vector machine and cross validation method were applied to assess classification accuracy. The classification performance of MIA methods (accuracy = 62.01%, precision = 62.03%, recall/sensitivity = 60.51%, and specificity = 82.80%) was much higher than classical methods (accuracy = 43.98%, precision = 43.81%, recall/sensitivity = 41.86%, and specificity = 70.50%), which extracted features contain similar energy based on a discrete wavelet transform, fractal dimension, and sample entropy. In this study, we found that emotion recognition is more associated with high frequency oscillations (51–100Hz) of EEG signals rather than low frequency oscillations (0.3–49Hz), and the significance of the frontal and temporal regions are higher than other regions. Such information has predictive power and may provide more insights into analyzing the multiscale information of high frequency oscillations in EEG signals.

## 1. Introduction

Emotion plays an important role in people’s daily life and cognition. Recently, emotion recognition has become a hot topic in the fields of brain-computer interface, artificial intelligence, and medical health, especially for the research and treatment of the mechanism and seizure law of diseases such as mental illness and psychological disorders [1]. However, emotion recognition based on the electroencephalogram (EEG) signals is still a well-known challenge.

Numerous new features have been investigated for emotion recognition based on the EEG signal analysis, including time domain, frequency domain, time-frequency domain, nonlinear analysis, and others. Frantzidis et al. [2] used event-related potential (P100, N100, N200, P200, and P300) as features in their study. Differential asymmetry, rational asymmetry, and power spectrum density are extracted as features for emotion recognition by Lin et al. [3]. Petrantonakis et al. [4] introduced higher-order crossing features to capture the oscillatory pattern of EEG. The Hjorth parameters are developed and used to distinguish emotions [5]. Liu et al. [6] proposed the fractal dimension (FD) based algorithm on quantification of basic emotions and described its implementation as feedback in 3D virtual environments. Several entropy-based metrics of signal complexity have already been proposed for discriminating emotional states. Hosseini et al. [7] applied two entropy metrics (approximate and wavelet entropy) to discriminate between two emotional states (calm-neutral and negative-excited) in response to viewing sequences of emotion-inducing pictures and achieved 73.25% classification accuracy. Jie et al. [8] applied sample entropy (SE) to EEG data obtained from two binary emotion recognition tasks (positive vs. negative emotion both with high arousal, and music clips with different arousal levels) and achieved 80.43% and 79.11% classification performance. Murugappan et al. [9] used the discrete wavelet transform (DWT) to divide the EEG signal into several bands. Then they calculated features based on these bands. Despite the fact that some encouraging progress has been made, developing the best combination of feature extraction and classification methods still require further research.

A promising development on EEG signals for emotion recognition is multiscale analysis, including correlation dimension, Lyapunov exponents, and entropy had been applied to investigate biological signals. In the time domain, Costa et al. [10] proposed the method multiscale sample entropy (MSE), which explored the interdependence between entropy and scale. In their case, the coarse-graining approach is used on different scales, which was widely used in EEG analysis [11], heart rate variability [12,13], and gait dynamics [14]. The complexity index calculated by the area under the MSE curve indicated the complexity of biological signals in different time scales [15]. Kostas et al. [16] applied MSE for emotion detection. In the frequency domain, there is another method to investigate multiscale information, i.e., empirical mode decomposition (EMD) [17] and its ensemble extension (EEMD) [18], which are fully data-driven, time-frequency techniques that decompose a signal into a finite set of amplitude/frequency modulated components, called intrinsic mode functions (IMFs). When EEMD applied to EEG signals, the entropy of each IMF can be calculated separately. Sharma et al. [19] used this method to identify focal EEG. Zhuang et al. [20] calculated the first difference of time series, the first difference of phase, and the normalized energy of IMF components for emotion recognition.

Entropy reflects the degree of disorder of the system. It can also be used to study the chaotic behavior of the brain. It is widely used in analyzing EEG signals since brain is a complex system. Several entropy-based metrics have already been proposed for estimating the complexity of signals. SE and Renyi entropy (RE) were applied to the automated diagnosis of epilepsy [21,22] and emotion [8,23]. Fuzzy entropy (FE) is highly sensitive to random information and insensitive to noise. Xiang et al. [24] used FE to detect epileptic seizure signals. FE based on EEG signals of the forehead area was also studied on driving fatigue [25].

Researchers have applied machine learning for emotion recognition using a dataset for emotion analysis using EEG, physiological and video signals (DEAP) [26]. The deep learning network and principal component analysis-based emotion recognition using the DEAP dataset has the accuracy rates of 52.03% ± 9.4 and 53.42% ± 9.4 for three levels (high, neutral, and low) arousal and valence classifications [27]. Mohammadi et al. [28] used DWT to decompose the EEG signal into several bands for extracting energy and entropy features and the classification accuracy is 86.75% for the arousal level and 84.05% for the valence level. Multivariate empirical mode decomposition-based feature extraction methods have been applied on the DEAP database, while artificial neural network yields the accuracy of 75% and 72.87% for arousal and valence states, respectively [29]. Although there are various methods for extracting emotion recognition features and some methods achieve high emotional recognition accuracy, there are still few attempts to apply multiscale information analysis (MIA). Previous studies on emotion recognition using the DEAP database mainly focus on two dimensions, i.e., arousal and valence, while few of them complete a classification in higher dimensions. Thus, in this study, we propose new methods for emotion recognition in four dimensions.

The aim of this study is to explore new features based on MIA of EEG signals for discriminating emotion states in four dimensions. In this study, the MIA methods are performed on a public emotion database DEAP. The area under the MSE curve of EEG signal illustrated the multiscale EEG complexity index (MECI) in the time domain. FE and energy based on EEMD evaluate the Multiscale Information of EEG signals in the frequency domain. The MECI, EEMD enhance energy and EEMD enhance FE constituted as a feature vector, which was fed into a support vector machine (SVM) classifier for emotional states classification. The proposed methods are compared with classical methods, which extracted features containing energy based on DWT, FD, and SE.

## 2. Materials and Methods 

### 2.1. Database

#### 2.1.1. Signals

The EEG signals used in this study were downloaded from the public database DEAP (http://www.eecs.qmul.ac.uk/mmv/datasets/deap/). The DEAP database includes EEG signals of 32 healthy participants (50 percent females), aged between 19 and 37 years old (mean age 26.9 ± 4.45). The experiment performed music video-induced emotion tasks, which presented 40 videos in 40 trials of each participant. During the experiment, EEG was recorded at a sampling rate of 512 Hz using 32 active AgCl electrodes (placed according to the international 10–20 system). They provided the data-preprocessed-matlab and data-original. The EEG signals of data-preprocessed-matlab were downsampled to 128 Hz. The electrooculography (EOG) artifacts were removed and a 4–45 Hz band-pass filter was applied. It is helpful for some researchers to analyze the preprocessed database, but we found that the EOG artifacts were not removed cleanly. The 128 Hz sample rate was so low that losing very useful information and the band-pass filter made it impossible to analyze the EEG signals at 45 to 100 Hz, which are meaningful for emotion recognition [30,31,32]. Therefore, we chose data-original to do the following analysis. Each trial in data-original is 60 seconds, and every subject has 40 trials. To increase the sample size, each 20-second signal was extracted as a sample, the data length of each sample is 10,240 (512 points/second × 20 second = 10,240 points) points. Therefore, there were 120 samples for each subject.

#### 2.1.2. Labels

**Figure 1:**
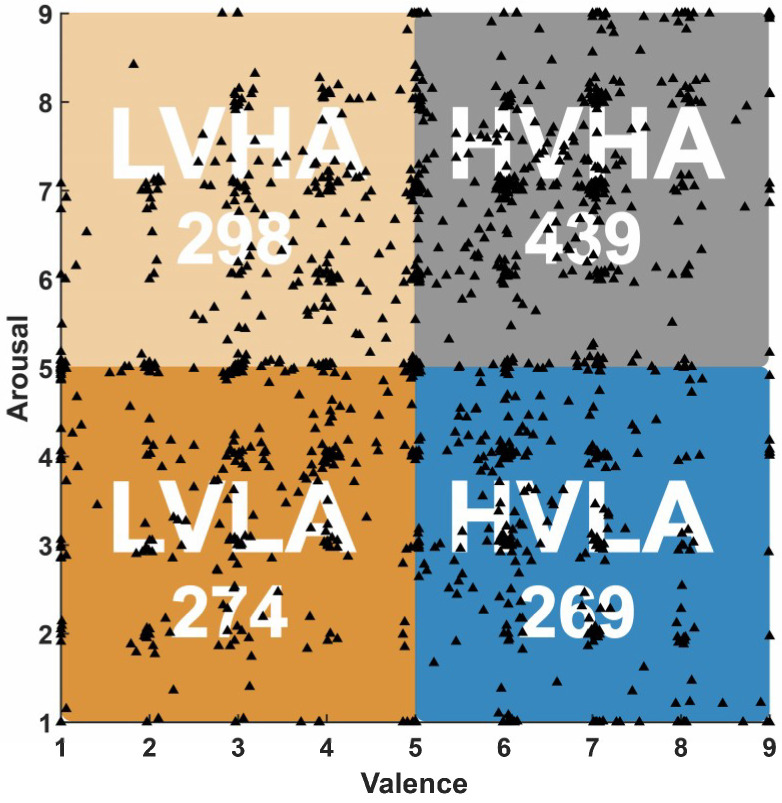
Emotion model classification. 439 high valence high arousal (HVHA) trials: valence > 5 and arousal > 5; 298 low valence high arousal (LVHA) trials: valence ≤ 5 and arousal > 5; 269 high valence low arousal (HVLA) trials: valence > 5 and arousal ≤ 5; 274 low valence low arousal (LVLA) trials: valence ≤ 5 and arousal ≤ 5.

At the end of each trial, the subjects performed a self-assessment task by providing the levels of valence, arousal, liking, and dominance. In this study, we took into consideration only the valence and arousal ratings. The self-assessment levels for valence and arousal ranged from 1 to 9. In this Russell’s circumplex model of the emotion model [33], the emotional states are characterized by two dimensions including valence and arousal and they can be mapped to a plane with arousal as the horizontal, and valence as the vertical axes. Arousal map emotions ranging from inactive to active while valence ranges from unpleasant to pleasant. In this study, we classified the emotion levels into four dimensions (see Figure 1). We can divide the 1280 trials of all participants into four-dimensional emotion groups based on the levels of valence and arousal, including 439 high valence high arousal (HVHA) trials, 298 low valence high arousal (LVHA) trials, 269 high valence low arousal (HVLA) trials, and 274 low valence low arousal (LVLA) trials. As shown in Figure 1, while valence > 5 and arousal > 5, it belongs to HVHA, while valence ≤ 5 and arousal > 5, belongs to LVHA, while valence > 5 and arousal ≤ 5, it belongs to HVLA, while valence ≤ 5 and arousal ≤ 5, it belongs to LVLA.

### 2.2. Methods.

#### 2.2.1. Data Preprocessing

Since we used the raw EEG data, it is necessary to remove all kinds of noises, especially EOG artifacts, which cannot be removed completely. To keep as much useful information as possible, we applied the 50 Hz notch filter and 0.3–100 Hz butterworth band-pass filter on the raw EEG data. EOG artifacts are often concentrated below 10 Hz [34]. Independent component analysis (ICA) [35] was used to remove EOG artifacts.

#### 2.2.2. Multiscale EEG Complexity in the Time Domain

In this study, to quantify the complexity of EEG signals in multiple time scales, multiscale EEG complexity index, i.e., MECI, was defined based on the MSE technique [10]. The process of MSE is as follows for time series *x_i_* = (*x*_1_, *x*_2_, *x*_3_, …, *x_N_*) *i* = 1, 2, 3, …, *N*:

- First, set different time scale *τ* from 1 to *s*.
- *x_i_* is divided into non-overlapping windows of equal length *M*.
  (1) $$M=\mathrm{int}\left(\frac{N}{\tau }\right)\;\tau =1,2,3,\ldots ,s,$$
  The average was calculated for each window, so a new time series was obtained.
  (2) $$x\left(\tau \right)=\left(\mathrm{mean}(x_{1},x_{2},x_{3},\ldots ,x_{\tau }\right),\mathrm{mean}(x_{\tau +1},x_{\tau +2},x_{\tau +3},\ldots ,x_{2*\tau }),\ldots )\;\tau =1,2,3,\ldots ,s,$$
- Above all, it is called coarse graining, and SE was then calculated for each coarse-graining time series in different scale factors.
  (3) MSE(τ)=SpEn(x(τ),m,r) τ=1,2,3,…,s,
  where SpEn denotes SE [36], *m* denotes the vector of length, and *r* denotes the tolerance of similarity.
- When all SE for time scale *τ* from 1 to *s* are calculated, the MSE(*τ*) series was the multiscale entropy of the original time series.

If time scale *τ* = 1, MSE is the SE of the original signal.

The MSE curve represents the SE in different time scales. The area under the MSE curve, which estimated the sum of SE values over the range of scales, is defined as the new feature MECI in this paper. The algorithm flow is:

- Obtain the MSE curve for all samples (32 channels, 20-second, *m* = 2, and *r* = 0.15) of each subject.
- Make a classification on each scale and find the range of scales that have higher accuracy.
- Calculate the area under the MSE curve of higher accuracy range as MECI.

#### 2.2.3. Multiscale Analysis Methods in Frequency Domain

##### Empirical Mode Decomposition and Ensemble Empirical Mode Decomposition

Because of the nonlinear and nonstationary characteristics of EEG signals, EMD [17] is a suitable method to decompose EEG data. EMD decomposes original signals into several IMFs. The process of EMD is as follows for time series *x_i_* = (*x*_1_, *x*_2_, *x*_3_, …, *x_N_*), *i* = 1, 2, 3, …, *N*:

- Obtain the upper envelope *U_i_* and lower envelope *L_i_* of the original signal *x_i_*.
- Then, calculate the mean envelope *M_i_* of the upper and lower envelope.
- Middle signal is obtained by subtracting the mean envelope from the original signals.
  (4) $$y_{i}=x_{i}-M_{i},$$
- Determine whether the middle signal satisfies the IMF-conditions.
  a) Throughout the data segment, the number of extreme points and the number of zero crossing points must be equal or not more than 1.
  b) The mean envelope of the upper and lower envelope at any data segments is 0, which means the upper and lower envelope is asymmetry.
- If *M_i_* satisfied the conditions, the *IMF* = *M_i_* and the new original data is obtained by subtracting the *IMF* from *x_i_*. Repeat step a to step d. If *M_i_* does not satisfy the conditions, the *M_i_* is the new original data and repeat step a to step d.
- Lastly, we get several IMFs $c_{i}^{j}$ *j* = 1, 2, 3, …, *m* and a remaining signal *r_i_*.
  (5) $$x_{i}=\sum_{j=1}^{m}c_{i}^{j}+r_{i},$$

However, EMD has the mode confusion and boundary artifact problems in some cases, so EEMD [18] was designed to alleviate the problem. EEMD is a noise-added method.

- The first, white noise of finite amplitude is added to the original.
- EMD is used to calculate IMFs.
- Repeat step a and b many times.
- When n-th noise is added, we calculate the average IMFs.
  (6) $$X_{i}=x_{i}+\epsilon_{i}=\sum_{j=1}^{m}C_{i}^{j}+r_{i},$$
  where *x_i_* is the original data, *ϵ_i_* is the random white noise, and $C_{i}^{j}=c_{i}^{j}+\epsilon_{i}$ represents the IMF obtained for the n-th noise observation.

##### Comparison of EMD and EEMD

**Figure 2:**
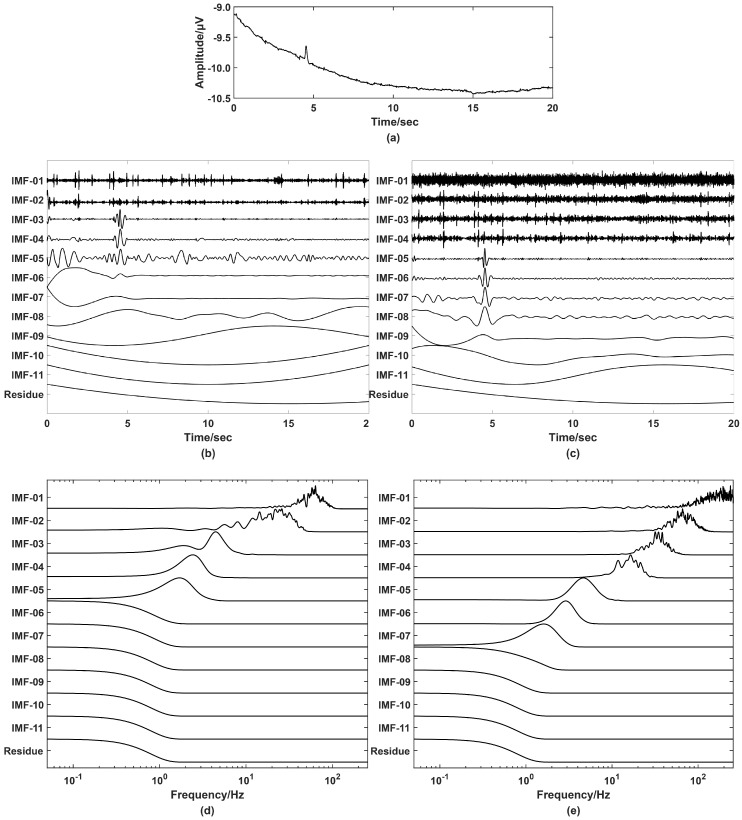
Comparison of results decomposed by empirical mode decomposition (EMD) and ensemble empirical mode decomposition (EEMD). (**a**) A 20-second raw electroencephalogram (EEG) signal without any preprocessing. (**b**) 11 intrinsic mode function (IMF) components and a residue decomposed by EMD on the EEG signals. (**c**) 11 IMF components and a residue decomposed by EEMD on the EEG signals. (**d**) Power spectral density (PSD) of IMF components and a residue decomposed by EMD. (**e**) PSD of IMF components and a residue decomposed by EEMD.

Since EMD and EEMD are both automatic decomposition methods, EOG artifacts can be decomposed into several IMFs, and then be removed accordingly. Therefore, in this study, raw EEG signals after 50 Hz notch filter were analyzed using EMD or EEMD. In this section, EMD and EEMD (noise ratio (standard deviation of the added white noise) is 0.1 and ensemble 100 times) were applied on a 20-second sample of raw EEG signal separately and shown in Figure 2. The 20-second EEG signal was decomposed into 11 IMF components, i.e., IMF1, IMF2, …, IMF11, and residue. The number of IMF = fix(log2(N))-1, where N is the length of input data. In this paper, data length is 10,240 points. According to the formula, there are 13 IMF components. However, as we can see in Figure 2, the frequency of the last few components are lower than 1 Hz. Therefore, the number is determined to be 11 IMF components and 1 residue after observing the decomposed results of a different number of IMF components. These IMF components can be divided into three categories.

- Clean signals: they have no EOG artifacts, baseline drift, head movement artifacts, or other obvious artifacts.
- EOG affected signals: they have clear EOG artifacts and head movement artifacts, but no baseline drift.
- Baseline signals: they are low-frequency baselines.

There is a clear EOG artifact at about 4.5 s in Figure 2a. The EEG signal was decomposed into 11 IMF components by EMD and EEMD separately and shown in Figure 2b,c. We found that, in Figure 2b, (1) IMF1 and IMF2 are clean signals and have no EOG artifacts, (2) IMF3 ~ IMF5 are EOG affected signals, and (3) IMF6 – IMF11 and residue are baseline signals. Figure 2c showed the IMF components decomposed by EEMD: (1) IMF1~IMF4 are clean signals, (2) IMF5~IMF8 are EOG affected signals, and (3) IMF9~IMF11 and the residue are baseline signals. The power spectral density (PSD) of all IMF components and a residue were calculated and shown in Figure 2d,e to discern the differences between EMD and EEMD. The frequency range of EEMD’s IMF1 covers from 75 to 256 Hz, which means this IMF has more white noise and high-frequency information of EEG signals. By observing the results of PSD, we found that the mode confusion problem is very serious when decomposing EEG signals with EMD compared with EEMD. Thus, we chose EEMD in the following analysis.

##### EEMD Enhanced Energy and Entropy

In this study, EEMD was applied to investigate multiscale information of EEG signals in the frequency domain, which decomposed the EEG signal into a finite set of amplitude/frequency modulated IMFs. For the $\mathrm{IMFs}\left\{imf_{1},imf_{2},\ldots ,imf_{N-1},imf_{N}\right\}$, each $imf_{N}$ is a time series $x_{i}=\left(x_{1},x_{2},x_{3},\ldots ,x_{N}\right)\;i=1,2,3,\ldots ,N$. There are two ways to compute the multi-scale information in the frequency domain: energy and entropy of each frequency scale (i.e., IMF) or combined frequency scales (i.e., IMFs) can be calculated. In the following paper, we used IMF1–2 representing the combination of IMF1 and IMF2, which means adding IMF1 and IMF2. Similarly, IMF1–3 represents the combination of IMF1, IMF2, and IMF3.

The process of EEMD enhanced energy and entropy is:

(1) Decompose the EEG signals of all samples (32 channels, 20-second, noise ratio = 0.1 and ensemble 100 times) into several IMFs with a different frequency scale using EEMD.
(2) Compute the Energy and Entropy of each IMF.
  *a)*  Energy
    For an $imf_{N}=x_{i}=\left(x_{1},x_{2},x_{3},\ldots ,x_{N}\right)\;i=1,2,3,\ldots ,N$, the energy [37] *E_n_* is defined as follows:
    (7) $$E_{n}=\sum_{1}^{N}x_{n}^{2},$$
    When applying EEMD on EEG signals, EOG artifacts are not removed. We did not use the normalized energy of the IMF in this study.
    The entropies used in this paper include SE [36], FE [38], and RE [22]. We compared the three entropy-based methods applied on IMF components.
  *b)*  Sample Entropy
    Sample entropy is a modification of approximate entropy [36]. We have an $imf_{N}=x_{i}=\left(x_{1},x_{2},x_{3},\ldots ,x_{N}\right)\;i=1,2,3,\ldots ,N$ and use a time interval to reconstruct series $X_{m}\left(i\right)=\left(x_{i},x_{i+1},x_{i+2},\ldots ,x_{i+m-1}\right)\;i=1,2,3,\ldots ,N-m+1$. The length of sequence is *m*. The distance function of two sequences is $d\left[X_{m}\left(i\right),X_{m}\left(j\right)\right]$. For a given embedding dimension *m*, tolerance *r* and number of data points *N*, SE is expressed as:
    (8) $$\mathrm{SE}\left(r,m,N\right)=-\log\frac{A^{m+1}\left(r\right)}{B^{m}\left(r\right)},$$
    where, $B^{m}\left(r\right)$ is the number of template vector pairs having $d\left[X_{m}\left(i\right),X_{m}\left(j\right)\right]<r$ and represents the similarity between two sequences of length *m*, $A^{m+1}\left(r\right)$ is the number of template vector pairs having $d\left[X_{m+1}\left(i\right),X_{m+1}\left(j\right)\right]<r$ and represents the similarity between two sequences of length *m* + 1.
    The tolerance level *r* is usually set to a percentage of the standard deviation of the normalized data. For our case, we selected 0.15.
  *c)*  Fuzzy Entropy
    Fuzzy entropy [38] is the entropy of a fuzzy set, which loosely represents the information of uncertainty.
    For an $imf_{N}=x_{i}=\left(x_{1},x_{2},x_{3},\ldots ,x_{N}\right)\;i=1,2,3,\ldots ,N$, we reconstruct series with length *m*:
    (9) $$X_{m}\left(i\right)=\left(x_{i},x_{i+1},x_{i+2},\ldots ,x_{i+m-1}\right)-\frac{1}{m}\overset{m-1}{\underset{j=0}{\sum }}x\left(i+j\right)i=1,2,3,\ldots ,N-m+1,$$
    The distance function $d_{ij}^{m}$ of two sequences is $d\left[X_{m}\left(I\right),X_{m}\left(j\right)\right]$. Given *n* and *r*, calculate the similarity degree $D_{ij}^{m}$ through a fuzzy function $\mu \left(d_{ij}^{m},n,r\right)$.
    (10) $$\mu \left(d_{ij}^{m},n,r\right)=\exp\left(-{\left(d_{ij}^{m}\right)}^{n}/r\right),$$
    Define the function $\emptyset^{m}$ as
    (11) $$\emptyset^{m}\left(n,r\right)=\frac{1}{N-m}\overset{N-m}{\underset{i=1}{\sum }}\left(\frac{1}{N-m-1}\overset{N-m}{\underset{j=1,j\neq i}{\sum }}D_{ij}^{m}\right),$$
    $\emptyset^{m+1}\left(n,r\right)$ is got similarly. Lastly, the FuzzyEn(m,n,r) of the series is shown below.
    (12) $$\mathrm{FuzzyEn}\left(m,n,r\right)=\ln\emptyset^{m}\left(n,r\right)-\ln\ln\emptyset^{m+1}\left(n,r\right),$$
  *d)*  Renyi Entropy
    There is an $imf_{N}=x_{i}=\left(x_{1},x_{2},x_{3},\ldots ,x_{N}\right)\;i=1,2,3,\ldots ,N$ and that adopts n values with probabilities $p_{i}=\left(p_{1},p_{2},p_{3},\ldots ,p_{N}\right)\;i=1,2,3,\ldots ,N$.
    The Renyi entropy [22] of order α, where α≥0 and α=1, is defined as:
    (13) $$H_{\alpha }\left(p\right)=\frac{1}{1-\alpha }\log\overset{N}{\underset{i=1}{\sum }}p_{i}^{\alpha },$$
    We used the gaussian kernel to obtain the probability density function before calculating RE.
    (14) $$p\left(x\right)=\frac{1}{n\sigma }\overset{N}{\underset{i=1}{\sum }}k\left(\frac{x-x_{i}}{\sigma }\right),$$
    In this paper, we set α=2.
(3) Accumulate the IMFs one by one and compute the energy and entropy of combined IMFs.

#### 2.2.4. Support Vector Machine

**Figure 3:**
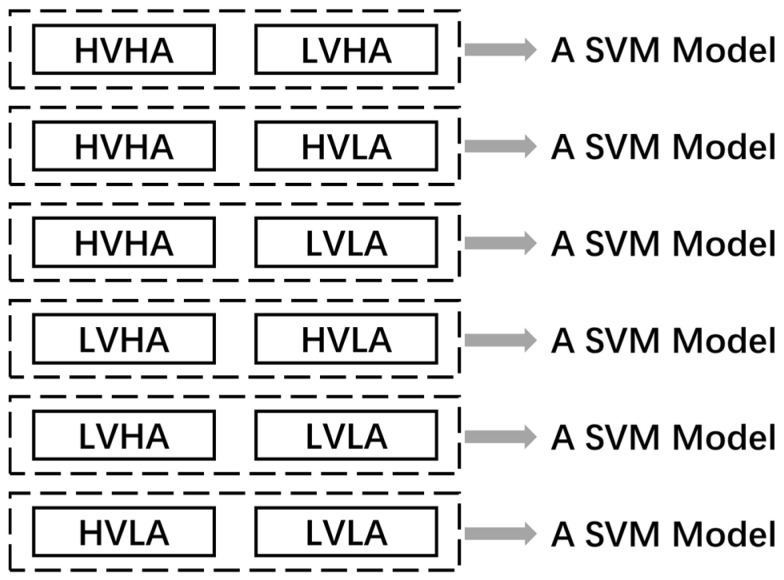
Architecture of multiclass support vector machine (SVM) classification with one-versus-one method.

The extracted features (MECI, EEMD enhanced energy and EEMD enhanced FE) were fed into SVM for classification. SVM is widely used for emotion recognition, which has promising properties in many fields. In our study, A library for support vector machines (LIBSVM) [39] is implemented for the SVM classifier with radial basis kernel function. The LIBSVM supports one-versus-one multi-classification, which was shown in Figure 3. If k is the number of classes, we generate k(k-1)/2 models, each of which involves only two classes of training data. In this study, six SVM models were generated for four-dimensional emotion recognition.

The 10-fold cross-validation was used before LIBSVM to divide the 120 samples into 10 parts. One of the 10 parts was used as a testing set and the remaining nine parts were used as a training set. To avoid information leakage, the samples from the same subject were divided into either training set or testing set. Before training, the features were normalized using function *scaleforSVM*, which process training and testing sets by mapping row minimum and maximum values to [−1, 1]. Then, the radial basis function kernel was selected, and the optimal parameters C and gamma were found by function *SVMcgForClass*. The mean value of 10-fold cross-validation will be used as the accuracy of this model.

#### 2.2.5. Statistical Methods

In this paper, the significance test was analyzed using Matlab R2018 (a). First, we tested whether the data satisfied the assumption of normality and the assumption of homogeneity of variance. We chose the parametric test ANOVA1, which is a one-way analysis of variance for the data that satisfied the assumptions. Otherwise, a nonparametric test Kruskal-Wallis was used. The significant difference was defined as the *p*-value < 0.05.

## 3. Results

### 3.1. Distinguishability of Emotional States in Four Dimensions Based on Time-Frequency Analysis

**Figure 4:**
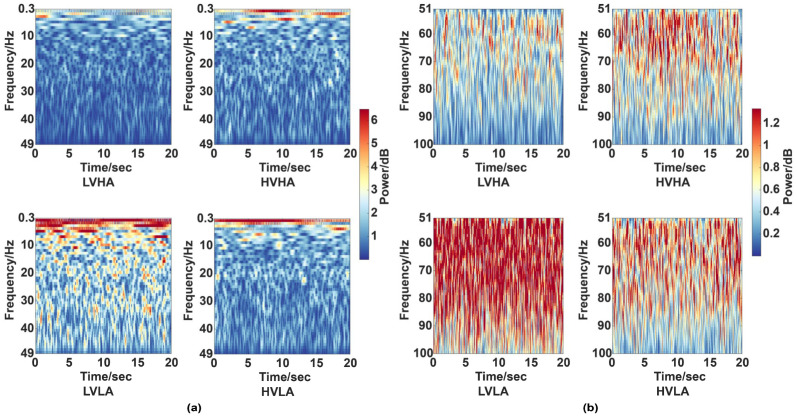
Time-frequency spectra of low frequency and high frequency oscillations. (**a**) Time-frequency images of four groups among low frequency oscillations (0.3–49 Hz). (**b**) Time-frequency images of high frequency oscillations (51–100 Hz). The color scales are shown in the right.

The difference in EEG oscillations related to emotions between the four groups (HVHA/LVHA/HVLA/LVLA) was explored. We divided the EEG signals into high frequency oscillations (51–100 Hz) and low frequency oscillations (0.3–49 Hz). The time-frequency analysis based on continuous wavelet transform (CWT) of EEG signals without EOG artifacts were analyzed and shown in Figure 4 (taking CZ of subject #32 as the example). Figure 4 indicated the more visible differences between four groups in high frequency oscillations rather than low frequency oscillations.

According to the observed phenomenon in Figure 4, it is reasonable to use high frequency oscillations of EEG signals for emotion recognition. The relationship between emotion and high frequency oscillations of EEG signals will be further verified below.

### 3.2. Multiscale EEG Complexity Analysis in the Time Domain

The complexity of EEG signals in different time scales contains different information. The multiscale EEG complexity was analyzed with MSE for emotion recognition. In this study, we used *m* = 2 and *r* = 0.15 for MSE, and 50 scales were calculated while covering 5.12–100 Hz (0.3–100 Hz band-pass filter was used during pre-processing). The relation between scale *τ* and frequency *f_τ_* is accorded with the following formula.

(15) $$f_{\tau }=\frac{f_{s}}{2*\tau },$$

where *f_s_* is the sample rate, in this study, *f_s_* = 512 Hz.

**Figure 5:**
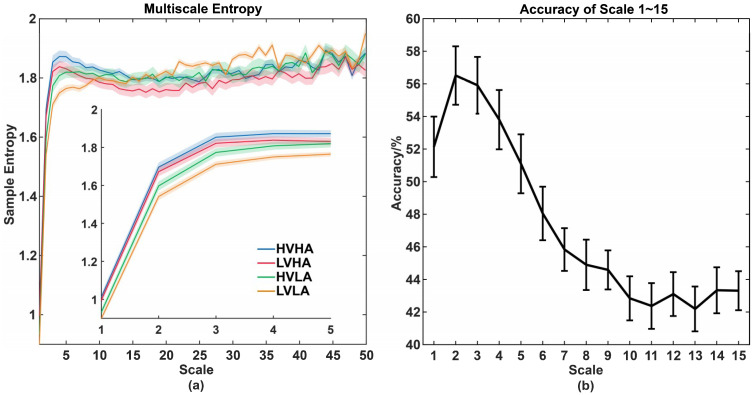
Multiscale sample entropy (MSE) curve of four groups and accuracy of scale 1–15. (**a**) The four curves represent the mean MSE curves (scale 1–50) of four-dimensional emotions for subject #32 and the shadow represents a standard error. The scale 1 to 5 was amplified. (**b**) Classification accuracy of scale 1–15 for all subjects.

We chose CZ of subject #32 as the example to compute the MSE curve from scale 1 to 50 and shown in Figure 5. From scale 15 to 50, the MSE curve of the four groups is interlaced with each other. The curves of HVHA and HVLA are almost coincident from scale 15 to 50. It indicated that there is no significant difference among the four groups from scale 15 to 50. On the other hand, the complexity curve at scale 1 to 15 has a relatively stable and distinct difference. The entropy of 32 channels on each scale (scale 1 to 15) was calculated as features and fed into an SVM classifier. Figure 5b summarized the classification accuracy of distinguishing emotional states in four dimensions for all subjects. The results presented that scale 1 to 5 have better performance where accuracy is higher than 50% and the best performance occurred at scale 2.

**Figure 6:**
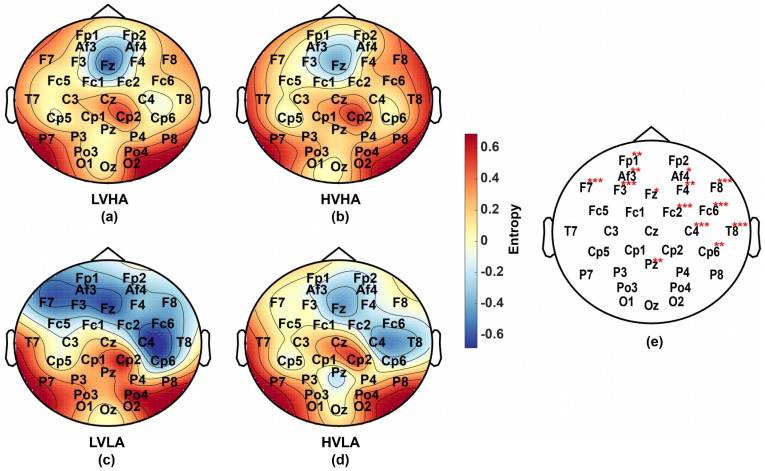
Brain map of complexity and statistical significance. (**a**)–(**d**) The brain maps of the averaged multiscale electroencephalogram complexity index (MECI) from scale 1 to 5 for subject #32: (**a**) Low valence high arousal (LVHA), (**b**) High valence high arousal (HVHA), (**c**) Low valence low arousal (LVLA), (**d**) High valence low arousal (HVLA). (**e**) The statistical significance of all subjects: 0.01 < Channel* < 0.05, 0.001 < Channel** < 0.01, Channel*** < 0.001.

According to the accuracies shown in Figure 5b, to ensure a wide range of adaptability and large differences, the MECI of scale 1–5 was calculated as a new feature of emotion recognition. According to formula (15) and 0.3–100 Hz band-pass preprocessing, the corresponding frequency range of scale 1 to 5 is 51–100 Hz, which is the high frequency oscillations of EEG signals. Then, the averaged MECI from scale 1 to 5 of subject #32 were calculated. Then the minimum and maximum values of the entropy were mapped to [−1, 1]. The results were displayed in Figure 6a–d, which include four different emotional groups. Figure 6e described the significant differences among the four groups (Kruskal-Wallis test) of all subjects and showed that the group differences in the frontal region and right-temporal region are much more significant than other regions. The results lead us to the conclusion that the relationship between emotion recognition and the high frequency oscillations of EEG is closer than the low frequency oscillations in the frontal region and the right temporal region.

### 3.3. Multiscale Information Analysis in Frequency Domain Based on EEMD

In this study, raw EEG signals of 32 channels (after 50 Hz notch filter) were analyzed using EEMD. EEG signals of each channel were decomposed into 11 IMF components by adding finite white noise (noise ratio is 0.1 and ensemble 100 times) to the investigated signal. The EEMD enhanced energy and entropy, which represent energy and entropy of each IMF and combined IMFs were extracted as features to explore the multiscale information of EEG signals in the frequency domain for emotion recognition.

#### 3.3.1. EEMD Enhanced Energy Analysis Based on the High Frequency EEG Oscillations

IMF1, IMF2, IMF3, and IMF4 were used to extract features, since the other IMFs were affected by EOG artifacts (shown in Figure 2c). The EEMD enhanced energy of all subjects were extracted and then fed the features into SVM for classification. All the details are shown in Table 1, and the IMF2 got the best performance of 53.88% for emotion recognition in four dimensions. The correlation coefficient and energy ratio between IMF2 and other components were calculated. The four group classification accuracy of IMF4 is only 43.05%, while its correlation coefficient with IMF2 is only 0.1290 (<0.2), which means weak correlations.

Among combined IMFs including IMF1-2, IMF1-3, IMF1-4, IMF2-3, and IMF2-4, we found that the accuracy increased along with the energy ratio. The accuracy of IMF1-2 is close to IMF2 and the correlation coefficient of IMF1-2 and IMF2 is 0.9510. IMF2 is approximate to the high frequency oscillations (51–100Hz, see Figure 2e). Hence, IMF2 contained the main information for emotion recognition in a frequency domain, which is consistent with the results of MIA in time domain described in Section 3.2, and we will use IMF2 in the following analysis.

#### 3.3.2. EEMD Enhanced Entropy Analysis Based on the High Frequency EEG Oscillations

In Section 3.3.1, it has been certified that IMF2 has the highest correlation with emotional states, so we compared effectiveness of SE, FE, and RE based on IMF2 for distinguishing emotions. For the sake of comparison in the same way, we analyzed IMF2 of all 32 channels for each subject, while the parameter for SE was set as *r* = 0.2, *m* = 2, the parameter for FE was set as *m* = 2, *r* = 0.15, *n* = 2, tau = 1, and the parameter for RE was set as *q* = 2.

**Figure 7:**
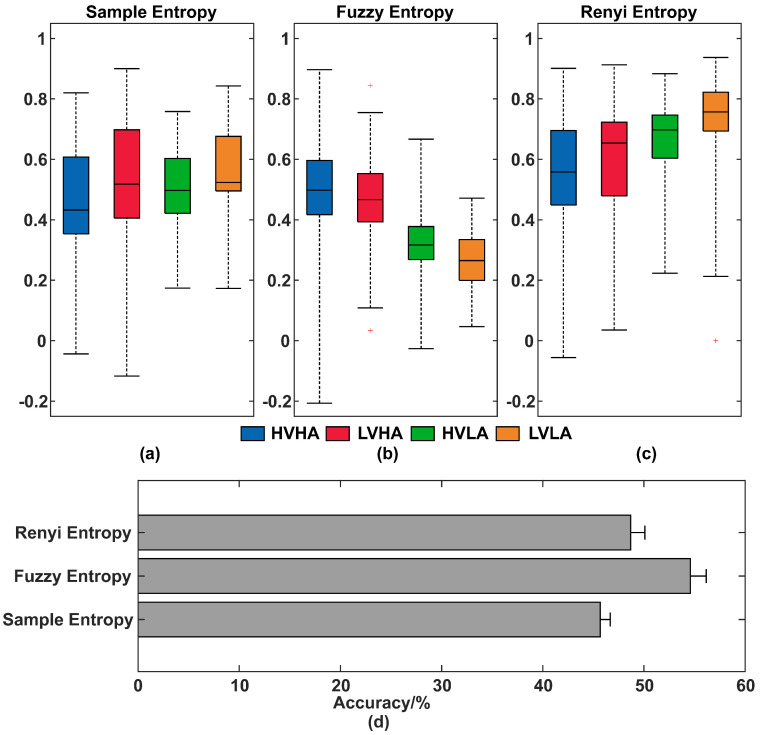
Comparison of sample entropy (SE), fuzzy entropy (FE), and renyi entropy (RE) based on intrinsic mode function 2 (IMF2) for discriminating four emotions. (**a**)–(**c**) Ensemble empirical mode decomposition (EEMD) enhanced entropy of four groups for subject #32: (**a**) EEMD enhanced SE, (**b**) EEMD enhanced FE, and (**c**) EEMD enhanced RE. (**d**) Accuracy of SE, FE, and RE with all subjects for distinguishing four-dimensional emotions.

The comparison result of SE, FE, and RE based on IMF2 for subject #32 were shown in Figure 7a–c. Since entropy values > 1 and are not normally distributed, we mapped the minimum and maximum values of the entropy to [0, 0.99]. Then the fisher Z-transformation was used to transform the sampling distribution of entropy so that it became normally distributed. Confidence bounds were calculated and then inverse fisher’s Z-transform was used to re-transform to obtain the entropy and confidence bounds. The significance of four dimensional emotions was calculated among SE (*p* = 7.63 × 10^−5^), FE (*p* = 3.62 × 10^−15^), and RE (*p* = 8.76 × 10^−3^).

Then SE, FE, and RE based on IMF2 of all channels for all subjects were extracted as features and then fed into SVM for four-dimension classification. The accuracy results were shown in Figure 7d. FE had the best performance in all classifications. The accuracy of EEMD-enhanced FE is 54.58% when distinguishing four-dimensional emotions (HVHA, LVHA, HVLA, and LVLA). According to previous research studies [38], FE is similar to the physical meanings of approximate entropy and SE. It measures the probability of the new model. The larger the FE, the greater the probability that the new model will generate and the more complex the sequence. In FE (see Figure 7b), high arousal group (including HVHA and LVHA) has higher entropy than low arousal group (including HVLA and LVLA). The LVLA group has the lowest entropy, while HVHA group has the highest entropy.

### 3.4. Comparison between Different Brain Regions

**Figure 8:**
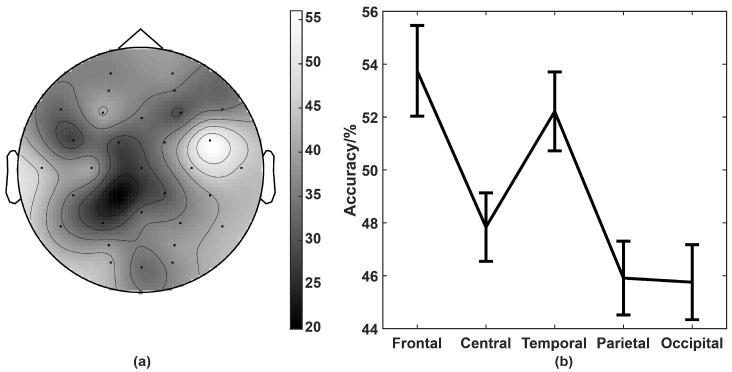
(**a**) Statistical significance of 32 channels, and (**b**) accuracy of five regions (frontal, central, temporal, parietal, and occipital). The brighter the place, the greater the difference.

The brain is divided into five brain regions, including frontal, central, temporal, parietal, and occipital regions, as shown in Figure 8. Three extracted features, including MECI, EEMD enhanced energy, and EEMD-enhanced FE were extracted as features for emotion recognition. The statistical differences (*p*-value) of 32 channels based on these three features were calculated for each subject. *N* is the number of three features for all subjects on 32 channels with *p*-value < 0.05 was counted. Figure 8a displayed that the brighter the channel, the larger the number *N*. We can see that the frontal and temporal regions are much brighter than in other regions. Then the classification accuracy of four-dimensional emotions in each region was calculated and shown in Figure 8b. Figure 8a,b have consistent results that frontal and temporal regions had the best performance. The results demonstrated that high frequency oscillations of EEG signals on frontal and temporal regions play an important role in emotion recognition.

### 3.5. Comparison of Multiscale Information Analysis Methods with Classical Methods 

In this part, we compared MIA methods (which extracted MECI, EEMD enhanced energy and EEMD enhanced FE as features) with classical methods (which extracted energy based on DWT, FD, and SE as features). We used box counting for FD calculating. The parameter for SE was set as *r* = 0.15, *m* = 2, and *N* = 512. The EEG signals were decomposed to several wave bands (delta band, theta band, alpha band, beta band and gamma band) by DWT based on “db4” wavelet. Then the energy of beta (16–32 Hz) and gamma (32–45 Hz) bands were calculated. We calculated the classification accuracy of all features with SVM. Table 2 showed that the accuracy of the proposed features (i.e., MECI (scale 1 to 5), EEMD enhanced energy, and EEMD enhanced FE) extracted using MIA is much higher than the features extracted by classical methods for four-dimensional classifications. MIA methods yield the highest accuracy 62.01%, while the accuracy of classical methods is 43.98%.

**Figure 9:**
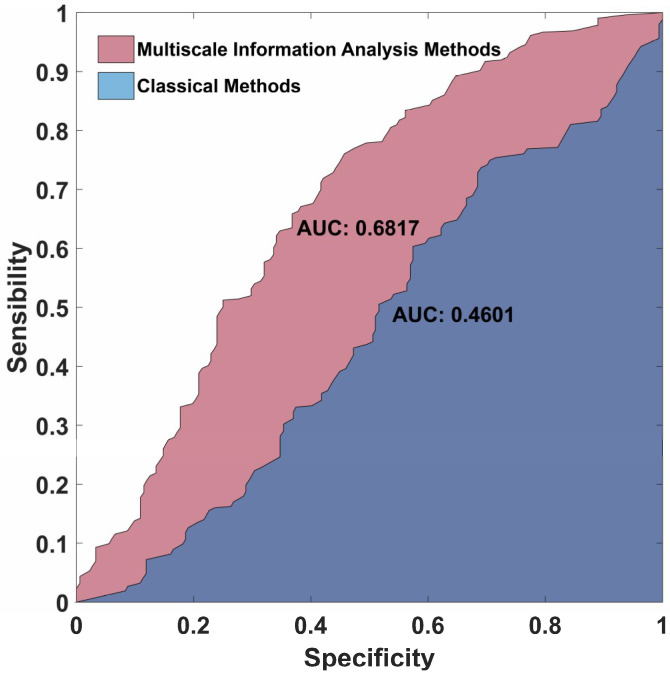
The receiver operating characteristic curve (ROC) of multiscale information analysis (MIA) methods and classical methods for subject #32. The area under curve (AUC) of MIA methods is 0.6817 and the AUC of classical methods is 0.4601, while the AUC of the reference line is 0.25.

**Figure 10:**
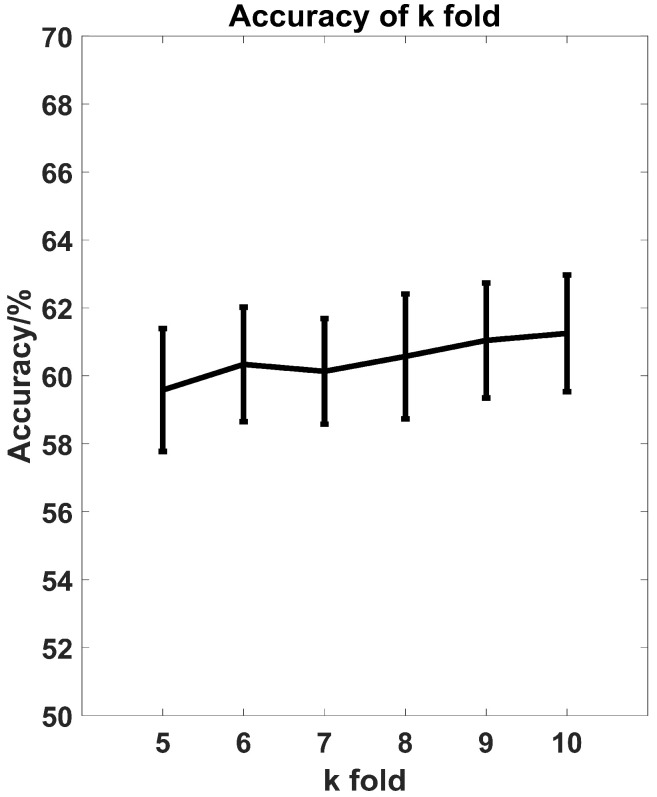
Accuracy of k fold (k = 5, 6, …, 10).

A receiver operating characteristic curve (ROC) is one of the important indicators to evaluate the performance of the model. In this paper, each participant has an independent SVM model. The ROC of subjects #32 was chosen and shown in Figure 9. The average ROC of four groups were computed as the ROC of the participant. As we can see in Figure 9, the area under curve (AUC) of MIA methods is 0.6817 and the AUC of classical methods is 0.4601, while the AUC of the reference line is 0.25 since there are four categories. Different folds (k = 5, 6, …, 10) of cross validation based on MIA methods were tested and the results were shown in Figure 10. As we can see that the accuracy has a slight improvement with the increase of folds, but it is basically the same, which means the 10-fold is sufficient for the classification of four emotional groups.

**Figure 11:**
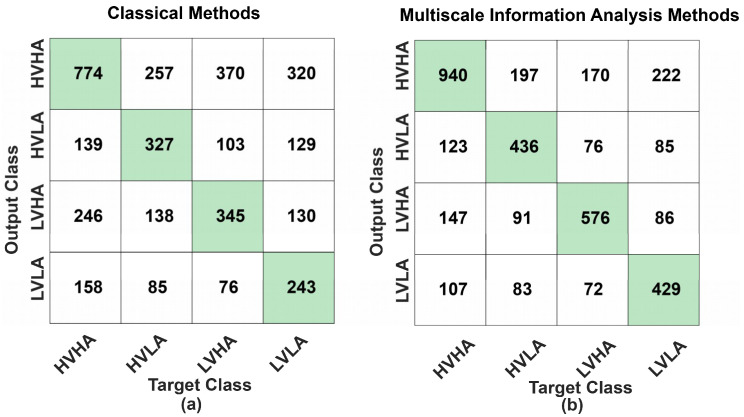
Support vector machine (SVM) polynomial kernel model confusion matrix. (**a**) Confusion matrix of classical methods. (**b**) Confusion matrix of multiscale information analysis (MIA) methods. The green grids represent the number of correctly predicted samples in each class, while the other grids represent the number of incorrectly predicted samples.

Confusion matrix, which contains precision, recall, sensitivity, and specificity is another method to judge the degree of classification besides the ROC curve and accuracy. The confusion matrix of classical methods and MIA methods for discriminating emotional states in four dimensions is calculated and presented in Figure 11 and Table 3. After classifier training, the remaining one-tenth of the data set (32 (subjects) × 120 (trials) × 10 (folds) = 38400 (samples)) is used for classifier validation. Each row of the matrix represents the instances in a predicted class while each column represents the instances in an actual class (or vice versa) [40]. As we can see, the precision, recall/sensitivity, and specificity of MIA methods were 62.03%, 60.51%, and 82.80%, and they were all higher than classical methods (precision = 43.81%, recall/sensitivity = 41.86%, and specificity = 70.50%). The results also indicated that high arousal including HVHA and LVHA are easier to recognize that low arousal including HVLA and LVLA.

## 4. Discussion

Emotion recognition based on EEG signals has achieved great progress in recent years. Many different kinds of methods in the time domain and frequency domain were proposed. In this paper, we presented the MIA methods to extract new features including MECI, EEMD enhanced energy, and EEMD enhanced FE. The results demonstrated that the proposed methods may help refine the effective information of EEG through MIA.

In recent years, emotion recognition based on DEAP mainly focused on binary classification instead of four-dimensional classification. Candra et al. [41] used wavelet analysis to recognize four-dimensional emotions and the sensitivity and specificity rates are 77.4% and 69.1%. However, they only picked up five subjects for training and another five subjects for testing from the whole 32 subjects, without any selection criteria. Wavelet energy, modified energy, wavelet entropy, and statistical features were studied by Ali et al. [42]. They also compared three different classifiers. The accuracy, precision, recall, and specificity of four-dimensional emotions based on the DEAP database are 83.37%, 62.53%, 61.96%, and 88.76%, which have high accuracy but low precision, and the deficiency is that they did not take the five to six scores into consideration when mapping the scales into four groups. Chen et al. [43] proposed a three-stage decision framework based on DEAP for distinguishing four-dimensional emotions. They achieved a high accuracy of 70.04%, while only 17 video trials, which have effective tags added by web users, were selected. Compared with the previous studies, the proposed features extracted using MIA from both time domain and frequency domain have improved the accuracy of emotion recognition (see Table 2 and Table 3).

It was claimed that emotion recognition has higher relativity with the high frequency oscillations of EEG than the low frequency oscillations. Li and Lu [30] indicated that gamma band (roughly 30–100 Hz) is suitable for EEG-based emotion classification. Müller et al. [31] found that a significant valence by hemisphere interaction emerged in the gamma band from 30 to 50 Hz. Jatupaiboon et al. [32] proposed a real-time EEG-based happiness detection system and the results showed that high-frequency oscillations (beta and gamma bands) give a better result than low-frequency oscillations. The same conclusion has been verified in this paper through time-frequency analysis and multiscale EEG complexity analysis in the time domain. We found that the recognition accuracy of the four-dimensional emotion levels based on high-frequency EEG oscillations (51–100Hz) is higher than that of low-frequency EEG oscillations (0.3–49 Hz).

In this paper, CWT was applied on time-frequency analysis, while there are some other time-frequency methods such as discrete fourier transform [44], discrete cosine transform [45], wave atom transform, and more [46]. Discrete fourier transform and discrete cosine transform based on the short term windowed analysis have the fixed window transformation problem, which leads to defects of spectrum analysis [47], while CWT enables more detailed analysis on signals.

Besides the standard MSE, which was used in this paper, a number of modifications and refinements of multiscale complexity were proposed and proved effective to the EEG signals with high frequency oscillations. There are generalized multiscale entropy [48,49], refined multiscale entropy, composite multiscale entropy [50], generalized multiscale Lempel–Ziv [51], and more, which were useful in quantifying the nonlinear dynamical complexity of the EEG series. Therefore, extension methods of multiscale complexity might be effective in emotional recognition.

In this study, EEMD was used to refine the frequency domain of EEG signals into multiple scales. Compared to fourier transform, EEMD can analyze non-linear and non-stationary signals, and, compared to wavelet transform, EEMD will not choose the basis function. However, EEMD does not fully solve the IMF mixing problem. Damaševičius et al. [52] proposed a novel noise cancellation method, which addressed the problem of mode mixing in EMD. If the problem of model mixing is solved and better decomposition results are obtained, the accuracy of emotion recognition may be further improved. Recently, entropy has become widely used in emotion recognition such as approximate entropy, SE, RE, FE, and Shannon Entropy. In this paper, the effect of FE based on EEMD is most outstanding for emotion recognition. The reason might be that FE is not sensitive to noise.

In order to compare the importance of different brain regions in emotion recognition based on MIA methods, we compared the significant differences between different regions. The frontal and temporal region is much more sensitive to emotions than other regions, which is reasonable because the frontal region participates in emotion regulation [40], while the temporal region can be activated by visual and auditory regulation [53].

Lastly, compared with classical methods, MIA methods have better performance (see Table 2 and Table 3). The viewpoint from the MIA proposed in this study might give a new way to distinguish different emotions. According to the results presented in this paper, the following conclusions could be addressed.

- The classification accuracy of four-dimensional emotion recognition is associated with the high frequency oscillations (51–100 Hz) of EEG than the low frequency oscillations (0.3–49 Hz).
- The frontal and temporal regions play much more important roles in emotion recognition than other regions.
- The performance of MIA methods is better than classical methods like energy based on DWT, FD, and SE.

However, some limitations have yet to be resolved. First, individuals have different performances of emotions on EEG signals. Dawson found that asymmetries in frontal EEG activity were associated with the type of emotion, while the generalized activation of frontal regions is associated with the intensity of emotion [54]. Thus, enough self-emotion reports and recorded signals as the training set is necessary for discriminating emotions. Furthermore, EEG can only detect electrical signals in the cerebral cortex, while emotion-related structures like amygdala [55] are buried below the cortex. Therefore, using EEG alone for emotion recognition is not enough. It is necessary to record other physiological signals and analyze synchronously to explore emotional states.

## Figures and Tables

**Table 1:** Comparison of performance for different intrinsic mode function (IMF) components selected for feature extraction (including 32 channels of 32 subjects) (Results are shown as mean ± standard deviation).

Components	Frequency Range (Hz)	Accuracy of Four Dimensional Emotions (%)	Correlation (IMF2)	Energy Ratio (IMF2)
IMF1	(64, 256)	43.96 ± 7.16	0.6932	7.1156
IMF2	(32, 128)	53.88 ± 11.04	1.0000	1.0000
IMF3	(16, 64)	46.20 ± 8.87	0.5447	0.4945
IMF4	(8, 32)	43.05 ± 7.74	0.1290	0.4511
IMF1-2	(32, 256)	52.81 ± 9.74	0.9510	0.6111
IMF1-3	(16, 256)	47.86 ± 7.92	0.8333	0.1751
IMF1-4	(8, 256)	47.55 ± 9.22	0.6600	0.0857
IMF2-3	(16, 128)	48.10 ± 9.66	0.7722	0.2251
IMF2-4	(8, 128)	47.32 ± 10.16	0.5704	0.1038

**Table 2:** Comparison of performance of all methods (Results are shown as mean ± standard deviation).

Methods	Features	Accuracy of Four Dimensional Emotions (%)	Accuracy of Four Dimensional Emotions Based on Combined Features (%)
Classical Methods	FD	39.51 ± 8.07	43.98 ± 8.88
	SE	42.42 ± 9.00	
	Energy of Beta	44.56 ± 8.49	
	Energy of Gamma	45.65 ± 10.00	
MIA Methods	MECI	53.46 ± 9.68	62.01 ± 10.27
	EEMD enhanced Energy	53.62 ± 10.80	
	EEMD enhance FE	53.70 ± 8.18	

**Table 3:** Evaluation indexes based on confusion matrix of classical methods and multiscale information analysis (MIA) methods.

Methods	Evaluations	HVHA	HVLA	LVHA	LVLA	Average
Classical Methods	Precision	44.97%	46.85%	40.16%	43.24%	43.81%
	Recall/Sensitivity	58.77%	40.52%	38.59%	29.56%	41.86%
	Specificity	49.14%	78.59%	72.34%	81.93%	70.50%
	Accuracy	——	——	——	——	43.98%
MIA Methods	Precision	61.48%	60.56%	64.00%	62.08%	62.03%
	Recall/Sensitivity	71.37%	54.03%	64.43%	52.19%	60.51%
	Specificity	70.99%	87.26%	84.78%	88.17%	82.80%
	Accuracy	——	——	——	——	62.01%
